# Molecular docking analysis of SARS-CoV-2 linked RNA dependent RNA polymerase (RdRp) with compounds from Plectranthus amboinicus

**DOI:** 10.6026/97320630017167

**Published:** 2021-01-31

**Authors:** Jayaraman Selvaraj, Umapathy Vidhya Rekha, Shazia Fathima JH, Venkatacalam Sivabalan, Rajagopal Ponnulakshmi, Veeraraghavan Vishnupriya, Malathi Kullappan, Radhika nalinakumari Sreekandan, Surapaneni Krishna Mohan

**Affiliations:** 1 Department of Biochemistry, Saveetha Dental College and Hospitals, Saveetha Institute of Medical and Technical Sciences, Saveetha University, Chennai - 600 077, India; 2 Department of Public Health Dentistry, Sree Balaji Dental College and Hospital, Pallikaranai, Chennai-600 100, India; 3 Department of Oral and Maxillofacial Pathology, Ragas Dental College and Hospitals, Chennai, India; 4 Department of Biochemistry, KSR Institute of Dental Sciences and Research, Thiruchengodu-637215, India; 5 Central Research Laboratory,Meenakshi Academy of Higher Education and Research (Deemed to be University), Chennai-600 078, India; 6 Department of Research, Panimalar Medical College Hospital & Research Institute, Varadharajapuram, Poonamallee, Chennai - 600 123, India; 7 Department of Clinical Skills & Simulation, Panimalar Medical College Hospital & Research Institute, Varadharajapuram, Poonamallee, Chennai - 600 123, India; 8 Department of Biochemistry and Department of Clinical Skills & Simulation, Department of Research, Panimalar Medical College Hospital & Research Institute, Varadharajapuram, Poonamallee, Chennai - 600 123, India

**Keywords:** SARS-CoV-2, RdRp, Plectranthus amboinicus, molecular docking

## Abstract

It is of interest to document the moelcular docking analysis of SARS-CoV-2 linked RNA dependent RNA polymerase (RdRp) with compounds from Plectranthus amboinicus. Hence, we report the binding features of rutin, Luteolin, Salvianolic acid A, Rosmarinic acid and p-Coumaric acid with the target protein SARS-CoV-2 linked RNA dependent RNA polymerase (RdRp) for further consideration.

## Background

The new strain of coronavirus SARS-CoV-2 (Severe Acute Respiratory Syndrome) is the infectious disease COVID-19 [1]. The structures of different SARS-CoV-2 protein / enzymes were solved. The structure data of RNA-dependent RNA polymerase (RdRp) and papa protease and key protease is relevant in drug discovery [2,3]. RdRp is the main enzyme that replicates the viral RNA genome and is it is a promising drug target [3-4]. RdRp of the SARS-CoV-2 shares 96 per cent of the sequence identity with SARS-CoV19 and hence the compounds or medications that are efficient towards RdRp of SARS-CoV are considered to be effective against the novel CoV. Molecular docking analysis of known RdRp-inhibiting antivirals, other FDA-approved medications, and phytochemicals to repurpose SARS-CoV-2 is documented [5]. The use of conventional medicines as an adjuvant for the treatment of COVID-19 is known [6,7,8]. Therefore, it is of interest to document the moelcular docking analysis of SARS-CoV-2 linked RNA dependent RNA polymerase (RdRp) with compounds from Plectranthus amboinicus.

## Materials and Methods:

### Protein Preparation:

The three-dimensional structure of the protein RdRp of SARS-CoV-2 (PDB ID: 6M71) was downloaded from the Protein Data Bank (www.rcsb.org/pdb). This structure is solved [10] with 2.90 Å resolution using electron microscopy. Three non-structured proteins (NSPs) such as one NSP7 and two NSP8 are involved in the structure as cofactors. NSP12, which is RdRp, is chain A and consists 851 amino acids. All water molecules, ions, and ligands were separated from the protein molecule using the PyMOL software. The hydrogen atoms were applied to the receptor molecule using the AutoDock Vina software's MG Tools [11] and saved in the Pdbqt format.

### Compound preparation:

Thirty compounds from the Plectranthus amboinicus plant were gleaned from literature. The compound structures were downloaded in .sdf format from the database of PubChem compounds (www.pubchem.ncbi.nlm.nih.gov/). All the compounds were translated to .Pdb format by using the online smiles converter. The energy of all ligands was minimized and translated to the PDBQT file format.

### Molecular docking and interaction analysis:

The grid box around the binding pocket is positioned using a standard protocol [12]. PyRx has been used to screen the ligand files against the protein [13]. The interactions between the targeted protein and the ligands were analysed using the Pymol Molecular Visualization Tools [14].

### Drug-likeness prediction:

The Lipinski filters (http:/www.scfbio-iitd.res.in / software / drugdesign / lipinski.jsp) were used to measure the drug likeness of the compounds from the docking calculation. Four of the five parameters defined for drug likeness are molecular mass, cLogP, hydrogen donor and acceptor and molar refractive index [14].

## Results and Discussion:

**Figure 1:**
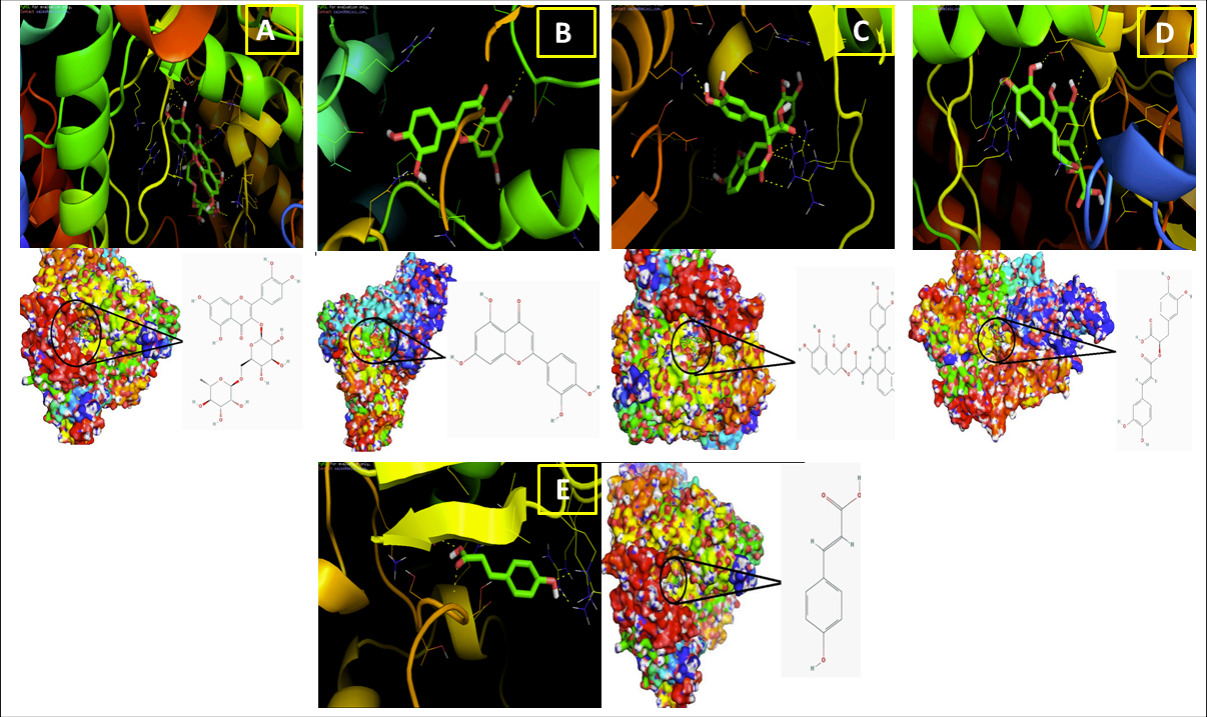
Molecular docking data of SARS-CoV-2 RdRp with (a) Rutin; (b) Luteolin; (c) Salvianolic acid A; (d) Rosmarinic acid and (e) p-Coumaric acid. Proteins are shown in ribbon and compounds are shown with stick representations

It is of interest to document the moelcular docking analysis of SARS-CoV-2 linked RNA dependent RNA polymerase (RdRp) with compounds (Table 1 - see PDF) from Plectranthus amboinicus. Hence, we report the binding features of rutin, Luteolin, Salvianolic acid A, Rosmarinic acid and p-Coumaric acid with the target protein SARS-CoV-2 linked RNA dependent RNA polymerase (RdRp) for further consideration (Table 2 - see PDf). The interactions between the targeted protein and the ligands were analysed using the Pymol Molecular Visualization Tools as shown in Figure 1.

## Conclusion

We report the binding features of rutin, Luteolin, Salvianolic acid A, Rosmarinic acid and p-Coumaric acid with the target protein SARS-CoV-2 linked RNA dependent RNA polymerase (RdRp) for further consideration.
